# Cases of Diseased and Wounded Arteries

**Published:** 1827-07

**Authors:** B. Travers

**Affiliations:** St. Thomas's Hospital


### DISEASED AND WOUNDED ARTERIES.

*Cases of Diseased and Wounded Arteries, treated principally at* St. Thomas's Hospital, *by* B. Travers, Esq. f.r.s.

**(Continued from vol. ii. p. 334.)**

*Case of Aneurism occurring in each Ham; the Disease showing itself in the Right Limb a few Weeks after the Cure of the Left; the Formation and Cure of both Aneurisms occupying a period of eight Months.*

Thomas Weale, æt. twenty-five, a mealman, accustomed to carry sacks of flour, admitted into St. Thomas's Hospital, November 14th, 1822. Seven weeks since perceived a painful pulsating swelling in the left ham, which has increased to the size of a swan's egg.

Nov. 22d.—The femoral artery was tied with a single ligature.

On the third day, the angles of the wound were found to have united by adhesion.

December 11th, (the nineteenth day after the operation,) the ligature came away. The sac was much reduced in size.

On the 2d of January, 1823, was discharged in good health.

He was re-admitted April 3d, with a pulsating tumor in the right ham, as large as a hen's egg, and exceedingly painful. About a week after his discharge, he was attacked with an intolerable itching in this ham, followed in a fortnight by violent cramps of the limb. Did not perceive the swelling till after the expiration of another fortnight, since which it has daily increased. The limb was placed in an easy position; he was directed to take Extract. Hyosc. gr. v. ter die.

April 11th.—The right femoral artery was tied with a single ligature.

By the third day, the wound had united by adhesion, as on the former occasion, except at the opening for the ligature, which came away on the thirteenth day; and on May 10th he was discharged in health, with good use of his legs.

In the Medico-Chirurgical Transactions, vol. ix. p. 409, is published a case of Aneurism, for which a second operation was performed with perfect success, (the ligature being placed immediately below the profunda,) six weeks after the failure of the first, in which the ligature had been removed at the expiration of twenty-seven hours.

In the same work, vol. vi. p. 656, is noted a preparation, taken from the body of a man whose external iliac artery was included in a ligature, having the epigastric artery above and the circumflexa ilii below it; and in whom secondary hemorrhage proved fatal, from the ulceration of the first-named vessel, at its angle of origin from the trunk, the circulation through the epigastric having continued.

A man, the subject of several aneurisms, died from gangrene of the operated limb, a few days after the operation. Another, the subject of an aneurism in each ham, died on the fourth evening succeeding the operation, of gangrene affecting the cellular membrane, which was discoloured, and distended with fetid gas as high as the loin. This man was aged thirty-two, of mid stature, florid complexion, light eyes and hair; had served twelve years in the army, during which time he had been engaged in several actions in the Peninsula, France, and Holland. On the evening of the operation, both feet were cold, and the limb operated upon, painful.

On the day following the operation, various livid spots appeared upon the foot, which was cold to the touch, and very painful. He had passed a restless night, and throughout the day had been fevered.

On the third day, livid spots appeared on the calf of the leg; the patient complained of severe pain in the head and abdomen, as well as in the affected limb, which, though sensibly colder than its fellow at and below the vesicated part, was as much warmer above that part. He had a very foul tongue, great nausea, a pulse ranging from 110 to 130, very small and feeble; a very pallid countenance, and anxious expression; great restlessness; slight delirium, and then stupor. His feces passed involuntarily, as soon as the bowels began to move. The lividity of the limb extended as far as the wound on the day of his dissolution.

The internal coat of the artery was inflamed for a considerable space above and below the ligature, but no lymph was effused. The arch and thoracic aorta presented many curd-like deposits upon their internal membrane, and a firm coagulum extended from the left ventricle throughout this portion of the aorta.

*A Case of Diffused Aneurismal Tumor from Wound of the Brachial Artery in Bleeding, cured by the application of a Ligature upon the Artery, above and below the Wound.*

August 20th, 1820.—Mary Bradford, æt. twenty, was bled from the arm, and the bleeding stopped in the usual way; but immediately after a swelling, having the appearance of a thrombus, was perceived, which in the course of two days increased to a very considerable size, extending from the elbow nearly to the wrist. There was much tension and throbbing pain, but no pulsation: the former had been partially relieved by the application of leeches and cold lotion.

22d.—This morning a copious hemorrhage took place from the lancet-wound: it produced syncope, and was then restrained. A free longitudinal incision being made into the tumor at the bend of the arm, and its contents, part fluid and in part coagulum, removed, the artery was exposed, and secured by two ligatures, including between them the wound, by which the vessel was nearly divided. The extensive cavity, at the bottom of which it lay, was fringed with lymph, and the natural appearance of the detached vessel much altered.

August 23d.—The arm is much less swollen, and is free from pain. Pulse 140. She has no appetite, but her rest is undisturbed. The incision has a sloughy appearance, and the original wound manifests an indisposition to heal.

Sumat Dec. Cinchonæ, Infus. Rosar. āā ℥j. sextis horis.—Applic[r] Lot. Acid. sub. Cataplasm Lini.

26th.—The integument between the two wounds has sloughed, and has been thrown off, leaving a healthy granulating surface. The sympathetic fever is much diminished. From this date the wound daily improved, and by the 14th of September cicatrisation was completed.

Sept. 18th.—Discharged in good health, with an useful arm.

*Division of the Posterior Tibial Artery, for which the Compress and Roller were tried without effect, and the Vessel was then secured by two Ligatures.*

August 19th, 1820.—John Heath, æt. forty-five, admitted with a wound an inch long in the back part of the leg, four inches above the ankle, inflicted by the scythe of a fellow mower. Profuse bleeding took place, till a bystander bound a handkerchief firmly round the limb, by which means it was stopped. On removing the handkerchief, hemorrhage recurred, and was arrested by the application of a tourniquet to the femoral artery. He was conveyed a distance of fourteen miles to the hospital. A sponge tent was introduced to the bottom of the wound, and a roller tightly applied from the foot to the ankle. The tourniquet was then slackened. After three hours, hemorrhage again took place, and the tourniquet was tightened.

21st.—The tourniquet slackened, without the recurrence of hemorrhage.

23d.—The sponge removed, and adhesive straps applied. The limb is rather swollen and painful; it starts occasionally. The patient has slight sympathetic fever.

24th.—Hemorrhage to the amount of twelve ounces in a full stream, stopped by the tourniquet.

25th.—The artery was cut down upon at the back of the tibia. The incision separated the lowermost fibres of the soleus muscle from their attachment, and took a direction indicated by the wound, being about four inches long: it exposed a cavity containing coagulum, and fringed with lymph. On scraping the surface with the knife-handle, the ends of the vessel, about an inch and a half asunder, were discovered, and tied each with a single ligature.

30th.—Both ligatures came away. The wound is healthy, and discharges freely.

September 21st.—Since the last report, the patient has been going on well. A slough was thrown off from the wound in the calf. A collection of matter has taken place above the heel.

28th.—The abscess communicates with the wound. Apply pressure above the heel.

October 4th.—Ulceration has taken place at the back of the heel, from which pus is freely discharged.

11th.—Another slough has been thrown off; the discharge is moderate.

27th.—Sits up.

November 12th.—Walks about with crutches.

January 31st, 1821.—Discharged in good health. The wounds have healed, and he has the perfect and full use of the leg.

*Remarks.*—Compression by the introduction of a sponge or other foreign body, is a mode of arresting hemorrhage which never can be with certainty depended upon, and therefore never should be had recourse to, when, as happens in almost all cases, the ligature can be applied. The permanent principle upon which it acts is that of stimulating to suppuration the fresh wound, the granulating surface of which effectually seals the bleeding orifice. Granulation is the quickest, if not the only process, by which lymph can be organised on the sides of a sac or cavity: while such a space is occupied by blood, or other effused fluid, granulation will not take place, although the presence of the fluid which is its proper secretion is not only favourable to its continuance, but necessary to its preservation. A sinus will very rarely granulate. A seton passed through it, or a stimulant injection, sometimes procures this object; but the destruction of the sinus by the knife sets its surface granulating immediately, and without fail. We refer to the case of a cavity or chamber, because in all wounds of arteries, shallow or deep-seated, this is formed by the blood which first issues, and this sac is large in proportion as bleedings have recurred and the vessel lies deep.

There are cases in which it is exceedingly difficult, if possible, to see the bleeding artery, as, after the removal of a tumor from beneath the lower jaw, &c. the blood is seen jetted rapidly from the bottom of the hollow, but not the vessel from which it is poured. There are cases in which a whole surface bleeds, and not one vessel of any magnitude can be detected. Sponge is the best substance that can be employed for arresting hemorrhage on this principle: it is easily introduced, swells and fixes itself, and, by the distention and irritation it produces, inflames the whole surface of the cavity. It should never be suffered to remain longer than two, or at the outside three days, but, like the suture, be invariably removed as soon as suppuration is established. The only objection to sponge is, that it becomes so fastened by the adhesive quality of the lymph deposited upon its cells and vessels shooting into it, that the presence of matter in abundance scarcely loosens it, so as to allow of its being easily removed. But, by poultices applied some hours before the attempt is made, and a little patience in the operation, its removal may always be accomplished without inducing bleeding. If sponge, or other foreign substance, is allowed to remain longer in the wound, it becomes such an irritant as to induce ulceration of the walls of the cavity, and then bleeding recurs; or to inflame the cellular membrane and absorbents of the limb, and give occasion both to neighbouring and remote abscesses. Thus a person to whose wounded radial artery sponge was applied, and improperly suffered to remain for several days, had an abscess which terminated in sloughing of the integuments of the hand, exposing all the extensor tendons; and, when this was healing, an abscess formed along the upper arm and axilla of the same side, by the discharges of which he was so exhausted as to sink under another accidental bleeding from a branch of the brachial artery, which was opened by ulceration.

A cavity such as we have described to be formed by the wound of an artery, is never more dangerous than when left to itself, as happens when the external wound has been small, and heals. This is a case complicated of spurious aneurism and abscess. The case of Ann Mould, (Medico-Chirurgical Transactions, vol. iv. p. 448,) is of this kind. The femoral artery had been divided by a carriage-wheel passing over the lower and back part of the thighs a fortnight before, and a cavity between the flexor muscles of the thigh formed, which, having gone into abscess, was for a time secure; but the ulcerative process being set up, and the wall of the abscess destroyed, the sealed mouth of the vessel was included in the destruction, and fatal hemorrhage ensued.

The writer of these remarks has known the sponge advantageously employed in several cases, both of wound and deep ulceration, in which, from its depth and concealment, the bleeding vessel could not be discovered after a long search; and also in bleedings from a surface, as in sloughing. He has seldom known it fail of effecting the object perfectly, when it was removed on the second or third day. The agaric, formerly much in use, acts, when retained, on the principle of the sponge.

Another principle of arresting hemorrhage is by directly producing slough,—i. e. disorganising the part by the cautery or escharotics. Of the last, the caustic potass and strong nitric acid are most in use.

The actual cautery, as it is called, is a very valuable instrument in some rare cases. The reader may find one recorded in a note to the paper before quoted, (Med.-Chirurg. Transactions, vol. vi. p. 657,) where the man's life (at present the driver of a hackney-coach in this town,) was undoubtedly saved by it. As in this case, a diseased artery which is insusceptible of the adhesive inflammation, is perhaps the best apology for its use. The most destructive hemorrhages follow the casting-off of sloughs, so that the adoption of this process for the prevention of hemorrhage seems to imply a paradox. But this is explained by the difference between the constitutional and purely local disease, or gangrenous inflammation and sphacelus. In the former, which is an ulcerative action, the circumscription of the slough is, for obvious reasons, uncertain; but if a proper sphacelus, or determinate adhesive boundary, is formed, as generally happens in sloughs artificially produced, though not always in those which are spontaneous, the security of the process, and in fact the principle, is the same. The slough is the sponge, with this difference, and perhaps advantage, that its detachment is to be effected by nature, and, it may be concluded, without danger of hemorrhage, the line of separation being marked; for this line is alike the indication and proof that she is prepared for it.

